# Dual Role of Sp3 Transcription Factor as an Inducer of Apoptosis and a Marker of Tumour Aggressiveness

**DOI:** 10.1371/journal.pone.0004478

**Published:** 2009-02-12

**Authors:** Khadija Essafi-Benkhadir, Sébastien Grosso, Alexandre Puissant, Guillaume Robert, Makram Essafi, Marcel Deckert, Emmanuel Chamorey, Olivier Dassonville, Gérard Milano, Patrick Auberger, Gilles Pagès

**Affiliations:** 1 University of Nice-Sophia Antipolis, Institute of Developmental Biology and Cancer Research UMR CNRS 6543, Centre Antoine Lacassagne, Nice, France; 2 University of Nice-Sophia Antipolis, INSERM, U895, Cell Death Differentiation and Cancer Team, Equipe labellisée par la Ligue Nationale contre le Cancer, Faculty of Medicine, Nice, France; 3 University of Nice-Sophia Antipolis, INSERM, U576, Regulation of immunity and inflammatory reactions, Nice, France; 4 Statistic service, Centre Antoine Lacassagne, Nice, France; 5 Chirurgical service, Centre Antoine Lacassagne, Nice, France; 6 Oncopharmacology unit (EA 3836), Centre Antoine Lacassagne, Nice, France; University of Edinburgh, United Kingdom

## Abstract

**Background:**

The ambiguous role of transcription factor Sp3 for tumour progression is still debated since it was described as a transcriptional repressor or activator. Here we tried to decipher the molecular mechanisms implicated in Sp3 accumulation observed in aggressive tumours.

**Methodology:**

We generated normal and tumour cell lines conditionally expressing Sp3. Cell growth was analyzed in vitro and after inoculation in nude mice. Apoptosis was assessed by pan- caspase activity assays, by counting fragmented nuclei and by determination of caspase 9 cleavage. Gene expression was determined by quantitative PCR. Cleavage by different caspases was performed after in vitro translation of the Sp3 cDNA in the presence of [S^35^] labelled methionine. Different tumour cell lines and head and neck tumour samples were tested for the presence of Sp3 by western blots. Correlation between Sp3 expression and overall survival has been statistically determined.

**Principal Findings:**

Conditional over-expression of Sp3 induces apoptosis and modifies expression of genes implicated in the regulation of cell cycle and pro and anti apoptotic genes. Sp3 over-expression strongly reduces the development of tumours in nude mice confirming its pro-apoptotic potential in vivo. However, cells can survive to apoptosis through selective Sp3 cleavage by caspase. Sp3 induction in established tumours resulted in transient regression then progression. Progression coincides with re-accumulation of the full length form of Sp3. Sp3 is over-expressed in tumour cell lines of different origins. The presence of high levels of the full-length form of Sp3 indicates a poor prognosis for overall survival of patients with head and neck tumours.

**Conclusions:**

Full length Sp3 accumulation highlights bypass of tumour cell apoptotic capacities and is indicative of head and neck tumours aggressiveness.

## Introduction

Sp3 is a zinc finger transcription factor that belongs to the Sp transcription factor family comprising Sp1, 2 and 4. Sp3 can act as an activator or a repressor of transcription depending on sumoylation processes [1]. It is expressed as a full-length 110 kDa protein but internal initiation of translation results in shorter Sp3 isoforms [2]. The relationship between Sp1 and tumourigenesis depends on the expression of genes implicated in growth control, apoptosis resistance and angiogenesis [3]. Moreover, down-regulation of over-expressed Sp1 protein in human fibrosarcoma cell lines inhibits tumour formation [4]. Sp1 is over-expressed in different types of tumour [5]. A relationship between Sp3 and cancer has also been suggested in recent studies [6] but has not been fully addressed. The above data have demonstrated the importance of Sp1 and Sp3 for tumour formation. However, over-expression of Sp1 results in apoptosis in different cell types [7]. This is controversial since Sp1 and Sp3 protect cortical neurons against apoptosis [8] and induce genes implicated in survival [9]. Moreover, betulinic acid, a natural product with anti-tumour activity, induces apoptosis by degrading Sp1 and Sp3 [10]. These data encouraged us to address the role of Sp3 in apoptosis and to evaluate its prognosis value in highly aggressive head and neck tumours.

## Results

### Sp3 over-expression is detrimental for cell maintenance

**Figure 1:**
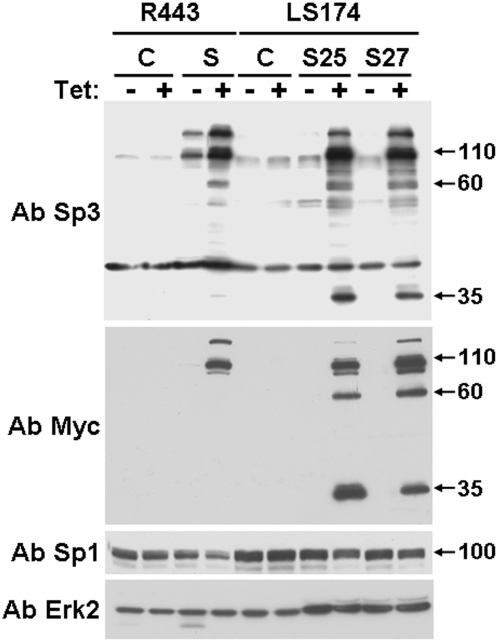
Western blot analysis of Sp3 expression in R 443 and LS 174 cells. Control R443 and LS174 cells (C) or their derivatives expressing Sp3 (S for R443 and S25 and S27 for LS174) were cultivated in the presence or absence of tetracycline for 24 hours and analysed for the presence of Sp3 by using Sp3- (Ab Sp3, D20 Santa Cruz, CA) and Myc-directed antibodies (Ab Myc, 9E10). The Sp1 levels were also analysed by using a Sp1 specific antibody (PEP-2, Santa Cruz, CA). Total Erk2 is shown as a loading control. This experiment is representative of three independent experiments.

**Figure 2:**
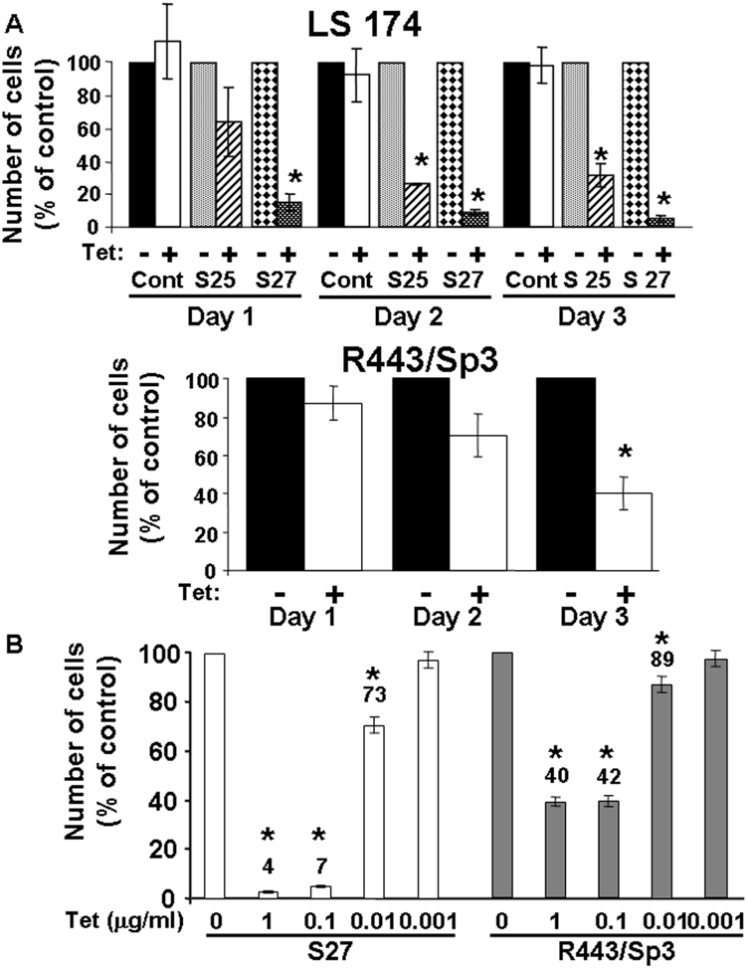
Inducible expression of Sp3 is detrimental for cell accumulation. A) Cells were cultivated in the presence or absence of tetracycline (1 µg/ml) and counted every day for three days. Each day, cell number in absence of tetracycline was taken as the reference value (100%). The percentage of cells remaining in the presence of tetracycline is plotted. For LS174 cells, two independent clones (S25 and S27) expressing different amounts of Sp3 were evaluated. For R443 cells, a previously described representative clone [11] was compared to control cells. These results are the mean of three independent experiments. Significant modifications (* p<0.05) are indicated. B) R443/Sp3 cells and LS174 cells (S27) were incubated in the absence (0) or in the presence of the indicated tetracycline concentrations for 72 hours. Cell amount in the absence of tetracycline was taken as the reference value (100%). The percentage of cells remaining in the presence of tetracycline is plotted. These results are the mean of three independent experiments. Significant modifications (* p<0.05) are indicated.

The role of Sp3 in apoptosis regulation was analysed in Chinese hamster lung fibroblasts (R443) and colon carcinoma cells (LS174) conditionally expressing full-length Myc-tagged Sp3 [11]. Both cells were chosen in order to determine the effect of Sp3 over-expression in a normal and a tumour cell line. Figure 1 shows that exogenous Sp3 was induced by tetracycline stimulation in the different cell types. Two independent LS174 clones (clone 25 (S25) and clone 27 (S27)) and one R443 clone conditionally expressing Sp3 were tested. Western blot experiments with an anti-Sp3 antibody showed that exogenous Sp3 was over-expressed compared to the endogenous form in both cell lines. Moreover, endogenous Sp3 was more highly expressed in LS174 than in R443 cells. Note that following over-expression, extra bands were detected at 60 and 35 kDa with both anti-Sp3 and anti-Myc antibodies. These bands were also observed in extracts from control LS174 cells after a longer exposure. Figure 1 also shows that overexpression of Sp3 do not significantly affect the Sp1 endogenous levels. Figure 2A shows that tetracycline induction of Sp3 resulted in decreased accumulation of both R443 and LS174 cells in a time dependent manner. Figure 2B shows that lower concentrations of tetracycline still induce cell death in R443 and S27 cells. The same effects were observed at concentrations of 1 or 0.1 µg/ml tetracycline since induction of Sp3 is equivalent at such concentrations as already described [11]. At a tetracycline concentration of 0.01 µg/ml Sp3 levels are significantly decreased while inexistent at 0.001 µg/ml.

### Sp3 over-expression induces apoptosis

**Figure 3:**
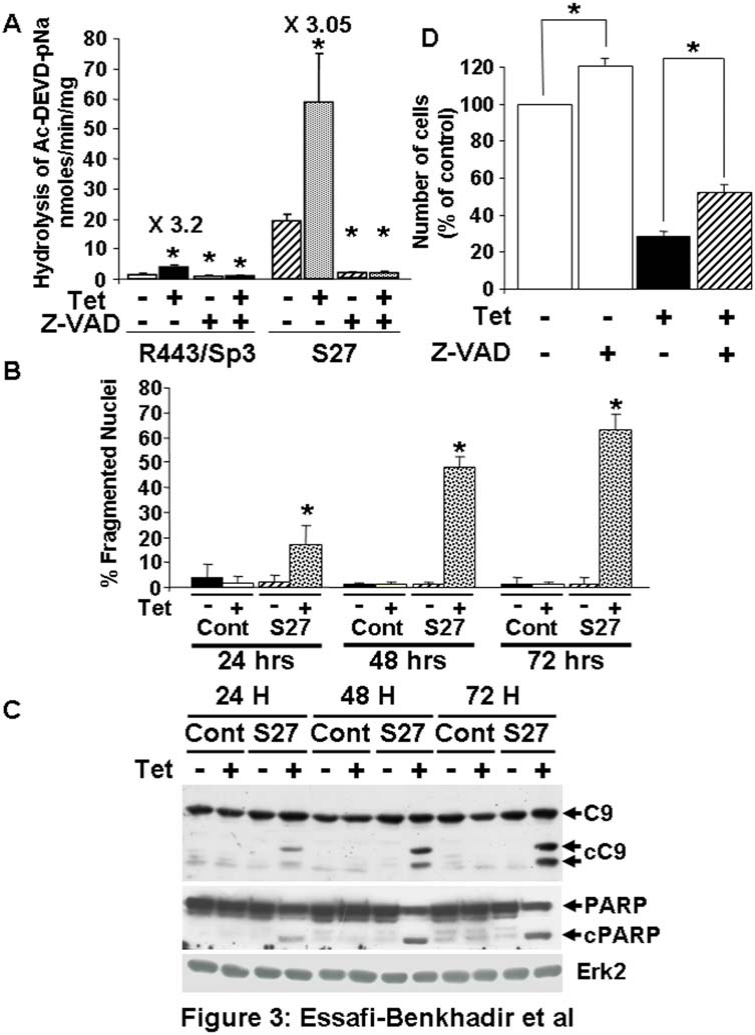
Sp3 over-expression induces apoptosis. Cells described in Figure 1 were tested for Sp3-mediated apoptosis by different methods. A) Pan-caspase activity was measured in R443 and LS174 cells expressing Sp3 (S27 for LS174) in the presence or absence of 50 µM Z-VAD-FMK as indicated in materials and methods. Results are the mean of three independent experiments performed in duplicate and significant modifications (* p<0.05) are indicated. B) Control or Sp3 expressing LS174 (S27) cells were cultivated in the absence (−Tet) or presence (+Tet) of tetracycline then stained with DAPI. At least ten photographs for each condition (twenty nuclei per photograph) were examined for the presence of fragmented nuclei. The percentage of cells with fragmented nuclei is plotted. The results are representative of three independent experiments. A significant increase of cell number with fragmented nuclei (* p<0.05) is indicated. C) Western blot analysis of caspase 9 (sc-7885, Santa Cruz, CA antibody) and PARP (C2-10, Santa Cruz, CA antibody) in LS174 or LS174 cells expressing Sp3 (S27) treated with (+) or without (−) tetracycline. The caspase 9 and PARP cleaved fragments (cC9 and cPARP respectively) are indicated by arrows. Erk2 is shown as a loading control. The results are representative of three independent experiments. D) LS174 cells expressing Sp3 (S27) were incubated overnight in the presence or absence of tetracycline (1 µg/ml) supplemented or not with 50 µM of Z-VAD-FMK. Cell number in absence of tetracycline and Z-VAD-FMK was taken as the reference value (100%). The percentage of cells remaining in the absence (−) or presence (+) of tetracycline supplemented or not with Z-VAD-FMK is plotted. These results are the mean of two independent experiments. Significant modifications (* p<0.05) are indicated.

Prevention of cell accumulation can be attributed to cell cycle arrest or induction of apoptosis. We carefully addressed the apoptosis mechanisms by three independent methods: measurement of pan-caspase activity, evaluation of the number of fragmented nuclei and cleavage of caspase 9. Figure 3A shows that induction of Sp3 resulted in increased pan-caspase activity. The specific pan-caspase inhibitor Z-VAD-FMK inhibits both basal and Sp3-dependent caspase activation which was stronger in LS174 cells (clone S27), probably reflecting the higher basal amounts of Sp3 in the absence of tetracycline. We have then more carefully addressed the apoptotic potential of Sp3 in the LS174 tumour cells. A quantitative analysis of fragmented nuclei upon Sp3 induction presented in Figure 3B illustrates that more than 50% cells were apoptotic following 72 hours of Sp3 induction and that the increased number of apoptotic cells paralleled the cleavage/activation of caspase 9 and PARP cleavage (Figure 3C), two substrates for active caspase 3 [12]. Sp3 effects on cell accumulation are partially inhibited by the pan-caspase inhibitor Z-VAD-FMK indicating that Sp3 induces a caspase-dependent apoptosis. Such reversion is coherent with the results of Deniaud et al who has observed the same effects of Z-VAD-FMK on Sp1-induced apoptosis [7]. Also note that Z-VAD-FMK treatment by itself results in cell accumulation suggesting that the basal caspase activity observed in figure 3A has been inhibited. Altogether our data clearly show that Sp3 over-expression is pro-apoptotic.

### Gene expression in Sp3-inducible LS174 cells

**Figure 4:**
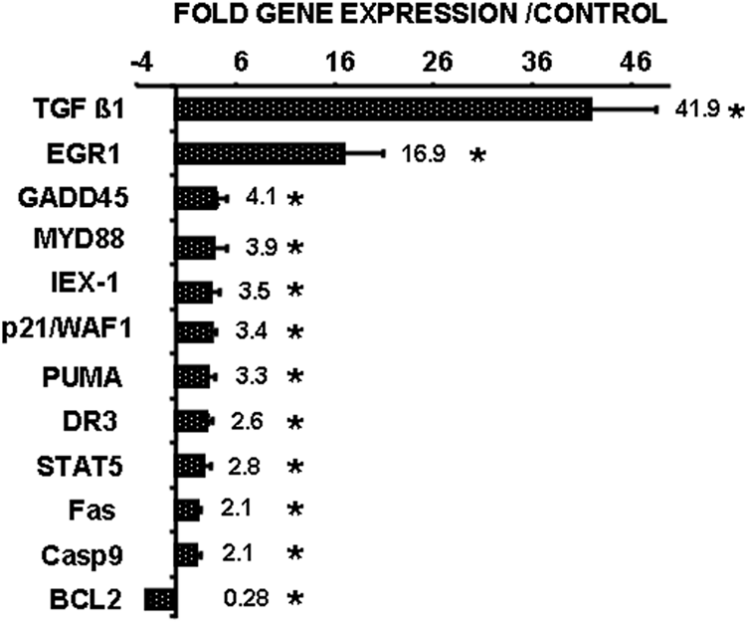
Gene expression profile in cells with conditional Sp3 expression. Expression of 88 pro- and anti-apoptotic genes was tested in LS174 cells conditionally expressing Sp3 (S27) by real time PCR. The variations of the significantly modified genes (* p<0.05) are represented. The mean fold increase±SD of three independent experiments performed in triplicate, is plotted.

The involvement of Sp3 in cell death was further evaluated by testing the expression of different specific transcripts involved in apoptosis and survival. We focused on a limited number of major pro- or anti-apoptotic genes (88) (File S1). Of these, 11 were up-regulated and only one (Bcl-2) was down-regulated. Figure 4 shows that both pro- (TGF β, GADD45, MyD88, DR3, Puma, Fas caspase 9) and anti-apoptotic (Egr-1, IEX-1, STAT-5, Bcl2) genes were modulated by Sp3. p21/WAF1 is not directly implicated in apoptosis but regulates cell cycle. Regulation of p21/WAF1 by Sp3 has already been described [13]. It suggests that the decrease in cell number observed in figure 2 is a combination of Sp3-induced apoptosis and cell cycle arrest. Since p27 expression is induced upon Sp3 down-regulation by siRNA [14] we have tested its amounts in both R443 and LS74 cells upon Sp3 induction. No modifications were observed in both cell lines (File S2). This complex set of regulated genes may explain the contradictory functions of Sp3 in the regulation of cell death and cell growth.

### Sp3 is a caspase substrate

**Figure 5:**
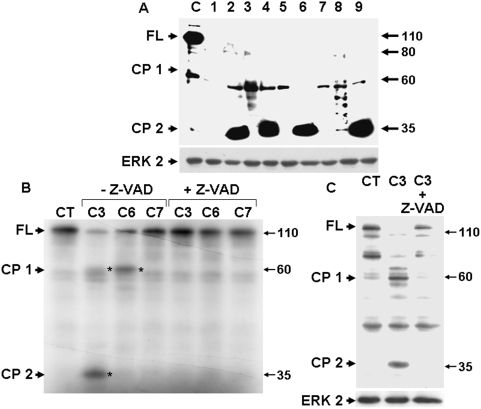
Sp3 is a caspase substrate. A) Analysis by western blot of the exogenous form of Sp3 in tetracycline-induced S27 cells (24 hours) and in clones chronically cultivated in the presence of tetracycline (1 to 9) with the Myc antibody. Arrows indicate the different reactive bands corresponding to the full-length protein (FL) or cleavage products (CP 1 and CP 2). Erk2 is shown as a loading control. This result is representative of two independent experiments. B) In vitro cleavage by caspase 3, 6 or 7 (BD Biosciences, Pharmingen) in the presence or absence of 50 µM Z-VAD-FMK of in vitro-translated Sp3. Cleavage products are indicated by asterisks. C) In vitro cleavage by caspase 3 in the presence or absence of 50 µM Z-VAD-FMK of Sp3 from tetracycline-stimulated LS174 cells (S27). The presence of cleaved products was analysed by western blot using an anti-Sp3 antibody. Cleaved products are indicated by asterisks. Erk2 is shown as a loading control.

After extensive apoptosis, a few cells became progressively resistant to Sp3 induction. Figure 5A shows the two main Sp3 degradation products of 60 and 35 kDa (cleaved product 1 and 2, (CP1, CP2)) detected with the Myc antibody in all the resistant clones. This suggests a cleavage mechanism at specific proteolytic sites. Note that both fragments were observed in control cells at a lower intensity, probably reflecting the high basal caspase activity of LS174 cells (Figure 1). In vitro cleavage assays on in vitro translated protein (Figure 5B) or on cell extracts from tetracycline-induced cells (Figure 5C) demonstrated that Sp3 is cleaved by caspase 3 and/or caspase 6 but not caspase 7. Such cleavage generates two fragments equivalent to those detected by western blot in control and tetracycline-resistant clones. Our results strongly suggest that Sp3 is a caspase substrate.

### Sp3 induction prevents tumour formation in nude mice

**Figure 6:**
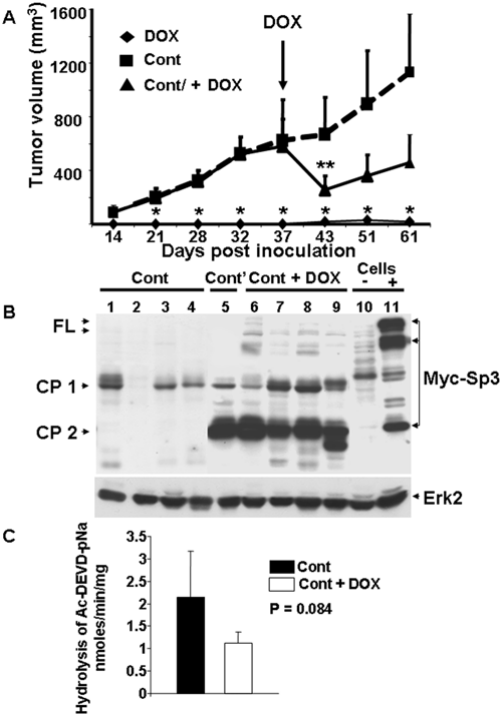
Sp3 prevents tumour formation in nude mice. A) Nude mice were inoculated with cells conditionally expressing Sp3 (LS174 clone S27). Comparison of the size of the tumours in animals receiving doxycycline in drinking water (DOX) or normal water (Cont). 37 days following inoculation, half of the control mice were treated with doxycycline (Cont+DOX). Tumour size was measured at the indicated times and tumour volume was calculated and plotted±SD. Statistically significant differences (p<0.05) between controls and doxycycline treated mice (*, immediate treatment after inoculation; **, treatment after development of the tumours) are indicated. B) Analysis by western blot with a Myc-directed antibody of extracts from tumours that have grown without tetracycline (Cont, lanes 1 to 4), a tumour that had grown in the presence of tetracycline (Cont', lane 5) and from tumours that had re-grown after tetracycline treatment of control tumours (Cont+DOX, lanes 6 to 9). Myc-tagged Sp3 expression was also analysed in S27 cells (Cells) treated (+, lane 11) or not (−, lane 10) by tetracycline. Arrows indicate the position of the full-length (FL) and cleaved Sp3 fragments (CP 1 and CP 2). Erk2 is shown as a loading control. C) Pan-caspase activity was measured in control tumours (Cont, black histogram) and in tumours that have grown in the presence of doxycycline (Cont+DOX, white histogram). Plotted is the specific caspase activity (total activity minus the values in the presence of 50 µM Z-VAD-FMK) measured as indicated in materials and methods. Results are the mean of three independent measurements of the individual tumour extracts.

LS174 cells conditionally expressing Sp3 were sub-cutaneously injected into nude mice. Figure 6A shows that injection of LS174 cells resulted in the formation of tumours two weeks following inoculation. Doxycycline-mediated induction of Sp3 prevented tumour development throughout the 60 days of follow-up. Only one mouse out of twelve developed a tumour upon doxycycline treatment. Western blot analysis revealed the absence of full-length Sp3 and the presence of Sp3 degradation products (Figure 6B, lane 5, Cont'). This suggests that Sp3-mediated cell death was bypassed by Sp3 cleavage. Interestingly, Sp3 induction in established tumours resulted in regression at first, but after two weeks progression was observed. Analysis of tumour extracts from tumours in the progression phase revealed the presence of Sp3 degradation products CP1 and CP2, but small amounts of exogenous full-length Sp3 were also detectable (Figure 6B, lanes 6 to 9, Cont+DOX). As expected, the full length form of Sp3 was undetectable in extracts from tumours that have grown in the absence of doxycycline (Figure 6B, lanes 1 to 4, Cont). Specific pan caspase activity in the control tumours and in the tumours that have grown in the presence of doxycycline (Figure 6C) is at least ten times lower than in the original cells (see figure 3A). Moreover, pan caspase activity has a trend to be superior in the control tumours than in tumours that have grown in the presence of doxycycline (Figure 6C, p = 0.084). These results suggest in vivo selection of mutant tumour cells with less caspase activity than the original cells. This lower caspase activity highlighted by Sp3 accumulation is a hallmark of aggressive tumour cells. In the adapted cells, full-length active Sp3 could stimulate transcription of genes involved in activation of the cell cycle [15] and angiogenesis [11]. Hence, the observed tumour progression could be the result of decrease apoptotic capacity and increased proliferation and angiogenic potential of the adapted cells.

### The presence of full-length Sp3 in head and neck tumours is an independent prognostic factor for overall survival

**Figure 7:**
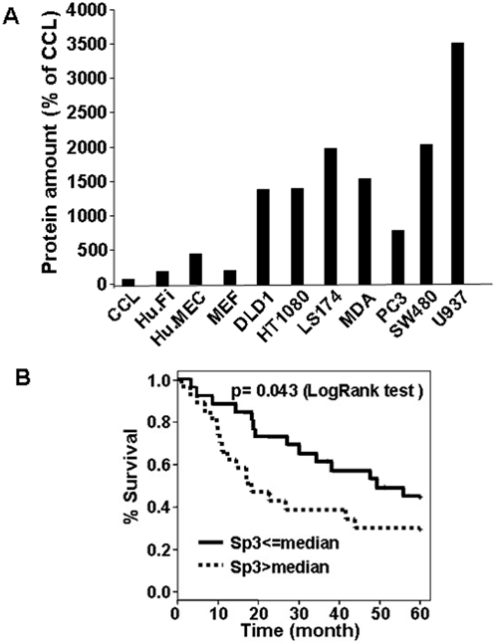
Sp3 is an independent prognosis factor for overall survival. A) Different normal (CCL, Hu.Fi, Hu.MEC, MEF) and tumour cells (DLD1, HT1080, LS174, MDA, PC3, SW480, U937) were analysed by western blot for their Sp3 content. The relative amounts of Sp3 are plotted. This experiment is representative of two independent experiments. B) Kaplan-Meier analysis of overall survival in patients with head and neck tumours in relation to their full-length Sp3 expression level (p = 0.043).

The relevance of Sp3 expression for tumour aggressiveness was first evaluated in different tumour cell lines. Sp3 was more highly expressed in tumour than in normal cell lines (fibroblast, endothelial cells) (Figure 7A). We then tried to extrapolate these results to human head and neck carcinomas (H&N). As in colon cancer, H&N tumours have mutations in ras and over-expression of EGF receptors leading to activation of the ERK pathway. We unsuccessfully tried to constitutively express Sp3 in H&N tumour cells, suggesting that the pro-apototic potential of Sp3 is also efficient in these cells. Sp3 levels in 52 H&N tumour samples were previously analysed by our team [16]. The median follow-up for all patients was 60 months. Table 1 and Figure 7B show that Sp3 expression is a factor of poor prognosis for overall survival (p = 0.043). Moreover, patient age, tumour size and nodal status were also significantly correlated with overall survival (p<0.05) and serve as positive controls. A multivariate Cox regression model showed that Sp3 expression is an independent prognosis parameter for overall survival (Table 2, p = 0.038). Western blot analysis on 16 tumours samples shows that Sp3 and Sp1 follow the same expression profile (low expression of Sp3/low expression of Sp1; high expression of Sp3/high expression of Sp1) which is consistent with the prognostic role of Sp1 described for other tumour types (File S3). It is interesting to note that the amount of CP2 in human and experimental tumours upon progression (Figure 6B) is more abundant than in cells stimulated by tetracycline for a few hours. This suggests that the phenomenon of adaptation has been induced progressively after a first round of degradation by caspases.

**Table 1:** Univariate analysis on overall survival of all patients according to age, clinical stage, nodal status and Sp3 expression.

Variable	Nb. of patients	Nb. of deaths	Median survival time (months)	p-value
**Patient age**				0.045*
≤70 years	46	27	41.7	
>70 years	6	5	9.1	
**Tumour size**				0.023*
T1–T2	16	6	Not reach	
T3–T4	36	26	21.1	
**Nodal status**				0.0083*
N0	16	5	Not reach	
N+	36	27	23.2	
**Sp3 expression**				0.043**
≤median	26	14	49.2	
>median	26	18	17.8	

* Log-Rank test.

** Peto and Peto modification of the Gehan-Wilcoxon test.

**Table 2:** Multivariate analysis (Cox Model) on overall survival of all patients.

Variable	Nb. of patients	HR	95% CI of HR	p-value
**Patient age**				
≤70 years	46	Ref.	Ref.	0.020
>70 years	6	1.19	[1.20–9.08]	
**Tumour size**				
T1–T2	16	Ref.	Ref.	0.13
T3–T4	36	0.70	[0.81–5.01]	
**Nodal status**				
N0	16	Ref.	Ref.	0.021
N+	36	1.15	[1.19–8.44]	
**Sp3 expression**				
≤median	26	Ref.	Ref.	0.038
>median	26	2.15	[1.04–4.42]	

HR: Hazard ratio.

CI: Confidence interval.

Ref.: Reference.

## Discussion

Identification of new prognostic markers for tumour aggressiveness is important for optimising treatments. For this reason we analysed the relevance of the transcription factor Sp3 as an indicator of tumour aggressiveness. Our study was based on previous observations describing Sp1 as an indicator of poor prognosis for pancreatic cancers [17]. However, the Shi et al study was contradicted by the results of Deniaud et al. [7], which showed that Sp1 over-expression induces apoptosis. Although it is over-expressed in tumour cells and tumour samples, Sp3 also induces apoptosis. One part of the puzzle has been elucidated since increased Sp3 expression seems progressive from benign to aggressive tumours [18]. The present data have highlighted that accumulation of Sp3 is progressive all along the tumour process. This mechanism of adaptation apparently depends on a balance between reduced of Sp3 cleavage and increased Sp3 expression and/or stability. Hence, the presence of the full-length active form of Sp3 results in increased transcription of many genes related to tumour growth [19] and angiogenesis [11]. The use of an inducible cellular system has allowed evaluation of the initiation of apoptosis related to Sp3 accumulation, a mechanism mimicking the first steps that potentially happen in vivo. In the case of tumour development, Sp3 could first be considered as a tumour suppressor during the initial steps, then as an oncogene, since it boosts growth and angiogenesis. Accumulation of Sp3 could be the result of increased ERKs activity since ERKs-induced Sp3 phosphorylation results in increased half-life [11]. The initiation of tumour development by oncogenes would allow adjustment of Sp3 levels, thus controlling the balance between growth and apoptosis. The latter (i.e. apoptosis) has been addressed by evaluating the genes activated upon Sp3 induction. Only 12 genes were modulated. While TGFβ [20], Egr1 [21], p21 [13], IEX1 [22], STAT5 [23], PUMA [24], FAS [25], BAK [26], Caspase 9 [27] and Bcl2 [28] contain active Sp3 binding sites in their promoters suggesting direct regulation, GADD45 (a target of p53 [29] and FOXO [30]) and MyD88 (a target of STAT-1 and IRF-1 [31]) do not, suggesting indirect regulation. Since TGFβ was maximally induced upon Sp3 overexpression and because its role on apoptosis was highly described, we tried to inhibit its action with blocking antibodies. No modification was observed suggesting that it is not the “master factor” implicated in Sp3-dependent apoptosis. The cell growth step of the mechanism has been addressed in two ways: 1) the possibility of obtaining cells resistant to Sp3-induced apoptosis through specific cleavage by caspase; the presence of a putative consensus cleavage site (DSSD) in the Sp3 sequence supports our results but the cleavage at this specific site needs to be confirmed; 2) formation of tumours in nude mice has highlighted in vivo selection processes. After an initial regression phase, a progression phase is observed. This progression coincides with an accumulation of the full-length form of Sp3. We hypothesised that the two phases observed in mice mimic the process which happens in human tumours resulting in *in vivo* selection of aggressive tumour cells with decrease apoptotic potential and increased growth and angiogenic capacities.

Our experiments have demonstrated the prognostic role of Sp3 in H&N tumours. Nevertheless, our study deserves to be extended to a greater number of samples and to different types of tumours.

## Materials and Methods

### Materials

Restriction and DNA-modifying enzymes and synthetic oligonucleotides were from Eurogentec (Liege, Belgium). Anti-Myc antibody (9E10), Anti-Sp1 (PEP-2) and Sp3 (D 20) were from Santa Cruz Biotechnology (Santa Cruz, CA). Anti-Erk 2 was a home-made antibody [32].

### Cell culture and transfection

Raf∶ER (R443) and LS174 cells conditionally expressing Sp3 were generated and cultivated as previously described [11]. CCL 39 cells (CCL, hamster fibroblasts), human fibroblasts (Hu.Fi), mouse embryo fibroblasts (MEF), DLD1 (colon adenocarcinoma), HT1080 (fibrosarcoma), MDA MB231 (metastatic breast cancer), PC3 (metastatic prostate cancer), SW480 (colon adenocarcinoma) were cultivated as LS174 cells. U937 cells (lymphoma) were cultivated in RPMI supplemented with 10% FCS, penicillin (50 units/ml), and streptomycine sulphate (50 µg/ml). Human microvascular endothelial cells (Hu.MEC) were kindly provided by Dr Ellen Van Obberghen-Schilling. For cell proliferation experiments, 5×10^4^ cells seeded in 12-well dishes were treated with/without tetracycline for the indicated times and counted using a Coulter counter (Beckman, Paris, France). Each value represents the mean of three replicates. Tetracycline was used at a final concentration of 1 µg/ml for each experiment.

### Preparation of RNA and gene array analysis by real-time quantitative PCR

1 µg of total RNA was reverse transcribed using random priming and Superscript II reverse transcriptase (Invitrogen). Real-time PCR was performed in an ABI PRISM 5700 Sequence Detector System (Applied Biosystems, Courtaboeuf, France) using the SYBR Green detection protocol and a dedicated “apoptosis plaque” (Applied Biosystems, Courtaboeuf, France) containing the major genes regulated upon apoptosis (see supporting information File S1). The relative expression level of target genes was normalized for RNA concentrations with four different house-keeping genes (GAPDH, β-actin, HPRT and ubiquitin). mRNA values are expressed as arbitrary units and represent the mean±SD of duplicates of two independent experiments. The sequences of the oligos corresponding to each gene tested in our study can be recovered on applied technology internet site (https://products.appliedbiosystems.com).

### Measurement of caspase activity

Each assay (in triplicate) was performed with 50 µg of protein prepared from control cells or cells stimulated with tetracycline as previously described [33]. Briefly, cellular extracts were incubated in a 96-well plate, with 0.2 mM of Ac-DEVD-pNa. Specific caspase activity was measured at 405 nm in the presence or the absence of 50 µM of the pan-caspase inhibitor Z-VAD-FMK. Caspase activity is expressed in nmoles of paranitroaniline released per min and per mg of protein. The same lysates were used for determining caspase 9 cleavage using a polyclonal antibody (sc-7885, Santa Cruz, CA) and poly (ADP-ribose) polymerase (PARP) using a monoclonal antibody (C2-10, Santa Cruz, CA).

### Measurement of fragmented nuclei

Cells were fixed with 5% paraformaldehyde in PBS (137 mM NaCl, 2.68 mM KCl, 1.47 mM KH_2_PO_4_, 8.1 mM Na_2_HPO_4_, pH 7.4). Morphological changes were observed using fluorescence microscopy under U excitation (330–380 nm) and photographed by a cooled CCD camera after staining with 0.5% (w/v) DAPI. For each condition, at least ten photographs were examined (twenty nuclei per photograph) and the number of fragmented versus entire nuclei recorded. The number of cells with fragmented nuclei was expressed as a percentage of total cells counted.

### In vitro caspase cleavage assays

Following subcloning of Sp3 cDNA into the pCDNA3 vector [11] it was translated in vitro and cleaved with recombinant caspases (BD PharMingen; San Diego, CA, USA) as previously described [34]. Long and short forms of Sp3 can be produced from this cDNA. The effect of the pan-caspase inhibitor Z-VAD-FMK (50 µM) was also monitored in vitro. The same protocol was applied to Sp3 obtained from cells stimulated by tetracycline for 24 hours. Following caspase treatment, proteins were run on 10% polyacrylamide gels.

### In vivo growth experiments and analysis of tumour parameters

Animal experiments were performed in accordance with the regulations of our institute's ethics committee. LS174 cells conditionally expressing Sp3 were injected subcutaneously into the flank of 6 week-old nude (nu/nu) mice (Harlan, Gannat, France). Doxycycline (750 µg/ml) was added to the drinking water from day 3 after the injection or when tumour volume reached an average of 600 mm^3^. Care of the animals was provided according to institutional guidelines: European Directive 86/609/EEC.

### Cell extracts and tumour sample analysis

50 µg of whole cell extracts from the different cell types or cytosolic extracts from tumour homogenates [16] were subjected to western blotting using anti-Sp3 (D 20, Santa Cruz, CA). The level of Sp3 in each cell or tumour sample was quantified with a GeneGnome imaging system (Syngene, Frederick, MD, USA) and normalised to the signals of β-actin and Sp3 present in LS174 cell extracts.

### Statistics

Quantitative data were analysed using one-way ANOVA. Data were expressed as means±SEM. P<0.05 was considered to be significant. For tumour analysis, all categorical data were described using numbers (n.) and percentages (%). Quantitative data were presented using median and range or mean and standard deviation. Censored data were described using Kaplan Meier estimation including median survival, and 95% confidence intervals (95% CI). Statistical analysis was two-sided and performed using R-2.5.0 for Windows. For univariate analysis, statistical comparisons were performed using the Chi-squared test or Fisher exact test for categorical data, the Student T test or Wilcoxon test for quantitative data, and Log-Rank test or Peto and Peto modification of the Gehan-Wilcoxon test for censored data. Multivariate analysis was performed by creating a Cox proportional hazards model. All variables associated with p<0.05 in the univariate analysis were included in the model. Proportional hazards tested for all entered variables using graphical (Schoenfeld residuals, log-log plot of cumulative hazard) and statistical methods. The search for interactions was automated using the R-2.5.0 stepAIC procedure, with interactions considered significant if p<0.01. Overall survival (OS) was defined as the interval between first diagnosis of cancer and death related to any cause. Follow-up was limited to 60 months for all patients. Patients lost to follow-up were censored after 60 months or at the date of their last known contact.

## Supporting Information

File S1 List of the investigated genes
(0.05 MB DOC)

File S2 Sp3 overexpression do not affect p27 expression. Control R443 and LS 174 cells (C) or their derivatives expressing Sp3 (S and S 27 for LS 174) were cultivated in the presence or absence of tetracycline for 24 hours and analysed for the presence of full-length form of Sp3 (FL Sp3) and p27 (Ref antibodies;K25020,Transduction Laboratories (Mississauga, ON,Canada)).Total Erk 2 is shown as a loading control. This experiment is representative of two independent experiments.
(0.91 MB TIF)

File S3 Expression of Sp3 and Sp1 in head and neck tumour samples. Analysis by western blot of tumour samples included in the statistical analysis. Arrows indicate the position of full-length or Sp3 cleaved fragments (FL, CP 1 and CP 2). Full length form of Sp1 (100 kDa) is indicated by arrows. Note the presence of Sp1 isoform of lower molecular weight. Actin is shown as a loading control.
(1.09 MB TIF)
