# Supplementary material for: Dual Role of Sp3 Transcription Factor as an Inducer of Apoptosis and a Marker of Tumour Aggressiveness
Source: PLoS One. 2009 Feb 12;4(2):e4478. doi: 10.1371/journal.pone.0004478 (PMC2636865; doi:10.1371/journal.pone.0004478)
Supplement: File S1 — List of the investigated genes (0.05 MB DOC) [file pone.0004478.s001.doc]

**Supporting information File 1:** List of the investigated genes

| Bad | BIRC8 | BCL2A1 | DR3 | Beta Actin |
| --- | --- | --- | --- | --- |
| Bak1 | BID | BCL2L2 | DR4 | GAPDH |
| Bax | TRADD | PAWR | DR5 | HPRT |
| BCL-X | DAP | DPF2 | DR6 |  |
| BCL-XL | SARP3 | cytochromC | TGF beta1 |  |
| BCL-XS | GAX | Smac/Diablo | Smad3 |  |
| BCL2 | Noxa | Apaf1 | Smad4 |  |
| Bik | PUMA | Hsp70 | STAT5 |  |
| Caspase 1 | SFRP1 | Hsp90 | EGR1 |  |
| Caspase 2 | Survivin | Bim | EGR2 |  |
| Caspase 3 | CFLAR/FLIP | MCL1 | EGR3 |  |
| Caspase 4 | CRADD | FADD | c-Myc |  |
| Caspase 5 | SCYE1 | IEX-1 | FKHR1 |  |
| Caspase 6 | FAIM | PDCD1 | FKHR2 |  |
| Caspase 7 | GBAG4 | PDCD2 | AFX |  |
| Caspase 8 | BCL10 | PDCD4 | GADD45 |  |
| Caspase 9 | DAPK3 | PDCD8 | p53 |  |
| Caspase 10 | DAXX | BNIP3 | p21/WAF1 |  |
| Caspase 12 | MDM4 | Fas | CARD11 |  |
| clAp1/BIRC2 | MYD88 | FasL | MALT1 |  |
| clAp2/BIRC3 | TP53BP2 | TRAIL | Moy HK |  |
| BIRC4 | DAP3 | TRAILR4 | Ubiquitin |  |
