# Supplementary figures and images for: Dual Role of Sp3 Transcription Factor as an Inducer of Apoptosis and a Marker of Tumour Aggressiveness
Source: PLoS One. 2009 Feb 12;4(2):e4478. doi: 10.1371/journal.pone.0004478 (PMC2636865; doi:10.1371/journal.pone.0004478)

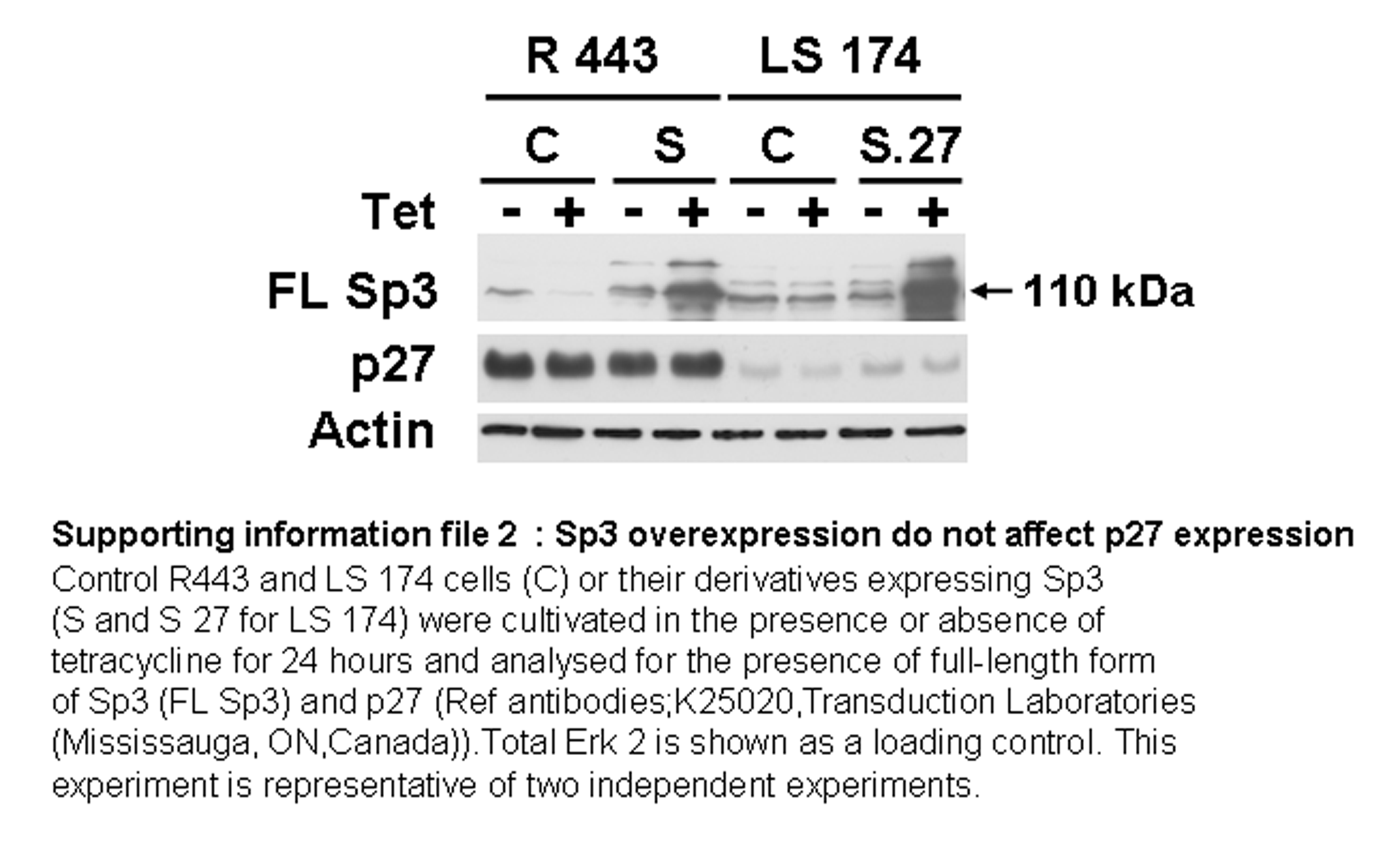

Supplement: File S2 — Sp3 overexpression do not affect p27 expression. Control R443 and LS 174 cells (C) or their derivatives expressing Sp3 (S and S 27 for LS 174) were cultivated in the presence or absence of tetracycline for 24 hours and analysed for the presence of full-length form of Sp3 (FL Sp3) and p27 (Ref antibodies;K25020,Transduction Laboratories (Mississauga, ON,Canada)).Total Erk 2 is shown as a loading control. This experiment is representative of two independent experiments. (0.91 MB TIF) [file pone.0004478.s002.tif]

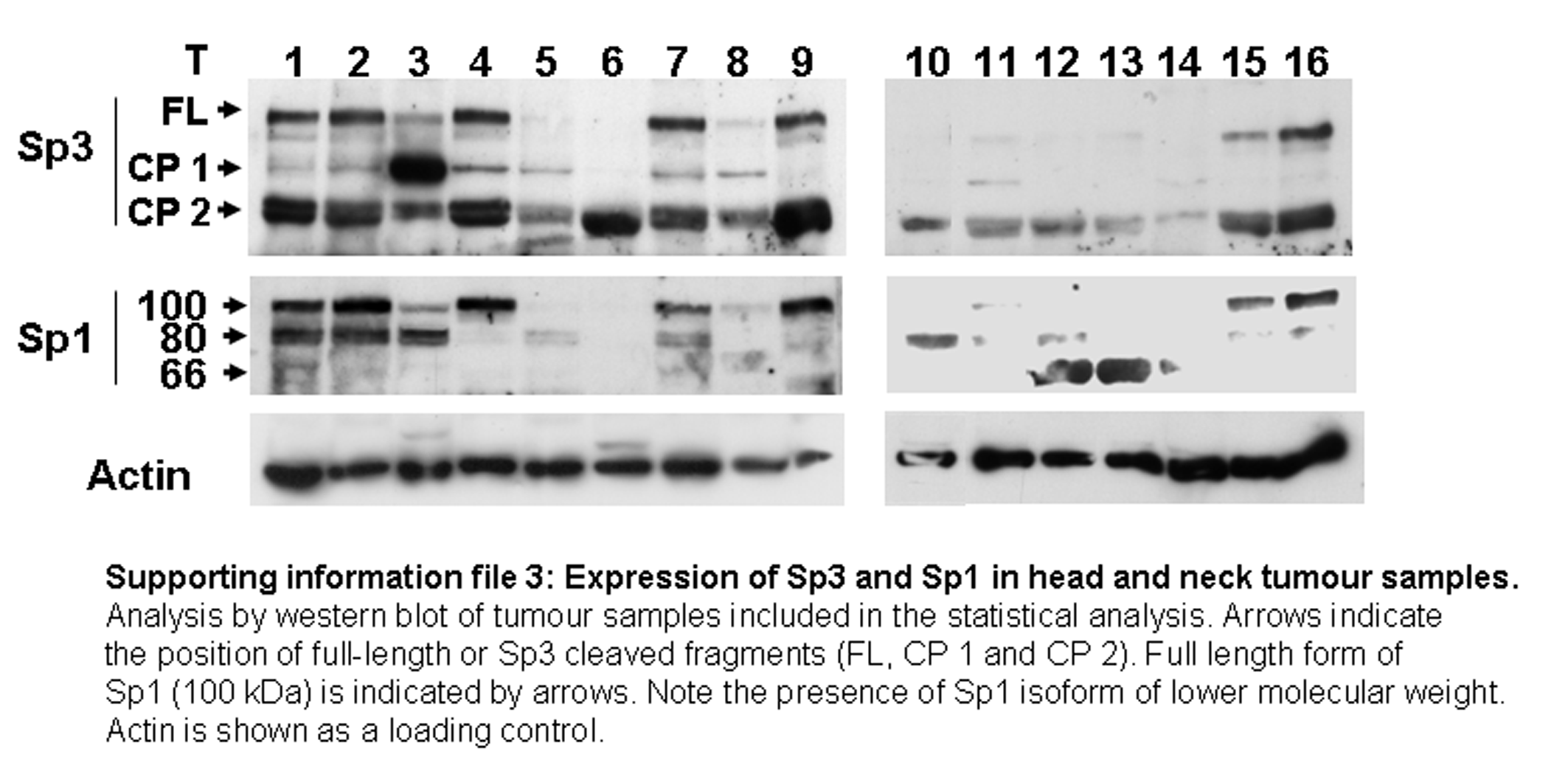

Supplement: File S3 — Expression of Sp3 and Sp1 in head and neck tumour samples. Analysis by western blot of tumour samples included in the statistical analysis. Arrows indicate the position of full-length or Sp3 cleaved fragments (FL, CP 1 and CP 2). Full length form of Sp1 (100 kDa) is indicated by arrows. Note the presence of Sp1 isoform of lower molecular weight. Actin is shown as a loading control. (1.09 MB TIF) [file pone.0004478.s003.tif]
